# Immediate initiation of antiretroviral treatment: knowledge, attitudes, and practices among clinic staff in New York City

**DOI:** 10.1186/s12913-023-09896-5

**Published:** 2023-09-28

**Authors:** Daniel Bertolino, Abigail Baim-Lance, Erica D’Aquila, Freda Coren, Bisrat Abraham

**Affiliations:** 1 New York City Department of Health and Mental Hygiene, Bureau of Hepatitis, HIV, and Sexual Transmitted Infections, Gotham Center, 42-09 28th Street, Long Island City, NY 11101 USA; 2 Brookdale Department of Geriatrics and Palliative Care, Icahn School of Medicine at Mount Sinai, New York, NY USA; 3 Institute for Implementation Science in Population Health, City University of New York, New York, NY USA; 4 Division of Infectious Diseases, Weill Cornell Medical College, New York, NY USA

**Keywords:** HIV, Rapid treatment, KAP, Mixed methods, ART, Antiretroviral treatment

## Abstract

**Background:**

Immediate initiation of antiretroviral treatment (iART) is a proven intervention that significantly decreases time to viral suppression and increases patient retention. iART involves starting medication as early as possible, often after a reactive rapid HIV test or re-engagement in care, although it does not have a universal definition. We aimed to understand iART from an implementation science perspective in a wide range of New York City (NYC) clinics providing HIV primary care, including staff knowledge, attitudes, and practices, as well as clinic barriers and facilitators to iART.

**Methods:**

We used a mixed-methods, convergent study design, with a quantitative survey and in-depth interview (IDI), to understand individual-level knowledge, attitudes, and practices, as well as clinic-level barriers and facilitators to iART. We recruited at least one medical and non-medical staff member from a diverse purposive sample of 30 NYC clinics. In quantitative analyses, we used separate binomial logistic regression models to estimate odds ratios (OR) and 95% confidence intervals (95% CI). In qualitative analyses, we used codebooks created by thematic analyses structured using a Framework Model to develop descriptive analytic memos.

**Results:**

Recruited staff completed 46 surveys and 17 IDIs. We found high levels of awareness of the viral suppression and retention in care benefits of iART. Survey respondents more commonly reported medication starts within three to four days of a reactive rapid HIV test rather than same-day initiation. Among survey respondents, compared to medical staff, non-medical staff were more likely to agree that medication should only be initiated after receiving confirmatory HIV test results (OR: 0.2, 95% CI: 0.06–0.8). Additionally, survey respondents from clinics serving a majority people of color were less likely to report iART on the same day as a reactive rapid HIV test (OR: 0.2, 95% CI: 0.02–1.0, p-value < 0.5). IDI results elucidated barriers to implementation, including perceived patient readiness, which potentially leads to added disparities in iART access.

**Conclusion:**

iART has proven benefits and support for its implementation among HIV clinic staff. Our findings indicate that barriers to expanding iART access may be overcome if implementation resources are allocated strategically, which can further progress towards health equity.

**Supplementary Information:**

The online version contains supplementary material available at 10.1186/s12913-023-09896-5.

## Background

In the United States, between 2016 and 2020 the overall rate of new HIV diagnoses consistently fell, with 30,635 new diagnoses reported in 2020 [1]. This trend coincided with HIV care standards evolving towards earlier initiation of antiretroviral treatment (ART) as evidence has shown this leads to reduced disease progression and improved overall population health [2–7]. Most recently, rapid or immediate initiation of ART (iART) has been shown to significantly decrease time to viral suppression (VS), increase patient retention, and increase VS durability, which in turn reduce HIV-related morbidity and mortality and onward HIV transmission [8–11]. Although iART does not have a universal definition, it involves starting medication as early as possible, including on the day of a reactive rapid HIV test (i.e., before receiving confirmatory laboratory results), soon after receiving confirmatory laboratory results, or on the same day a person with diagnosed HIV re-engages in care [12]. People who receive a reactive rapid HIV test still need their diagnosis to be confirmed, but they may begin ART in advance of this result [13]. This clinical practice aligns with the test and treat approach, which furthers progress towards attaining the UNAIDS 90-90-90 goals [14].

In New York City (NYC), an epicenter with an estimated 87,500 people with HIV as of December 31, 2021, efforts to combat the HIV epidemic, including through iART programs, resulted in new diagnoses dropping below 2,000 every year since 2018, unprecedently low numbers since annual reporting began in 2001 [15]. The NYC Department of Health and Mental Hygiene (DOHMH) has expanded access to iART in the city’s free sexual health clinics through its JumpstART Program, which from its launch in November of 2016 to November 2019, provided 640 New Yorkers immediate access to ART, with over 70% of these clients from priority populations (e.g., Black/African American or Hispanic/Latino men who have sex with men [MSM], women of color, transgender people) [16, 17]. Additionally, starting in 2018, NYC DOHMH incentivized iART among providers contracted through the Ryan White Part A Care Coordination Program and the Status Neutral Linkage and Navigation Services in Clinical Settings Program. To promote these programs among people with HIV, information on how to access iART services (i.e., program phone numbers, hours of services, and location) is publicly available online via the interactive NYC Health Map [18]. Despite declining diagnoses and increased access to ART, there are continued inequities in HIV acquisition and treatment in NYC and nationally [1, 15, 19]. For example, Black/African American and Hispanic/Latino residents of NYC made up 44% and 37% of people newly diagnosed with HIV in 2021, disproportionate with general population representation [15, 20]. Among all people newly diagnosed with HIV in NYC since 2017, 55% or less achieved VS within three months of their diagnosis each year, indicating limited access to iART and medication adherence support, and sustained population-level virus within disproportionately impacted communities [15].

Making iART more widely accessible has the potential to dramatically decrease onward disease transmission, reduce health care burdens placed on patients (e.g., requiring multiple visits prior to initiation of ART), and create more equitable health outcomes if implemented to scale [10, 21]. Whereas previous studies aimed to understand the health outcomes associated with iART, we aimed to understand iART from an implementation science perspective using an individual-level knowledge, attitudes, and practices (KAP) framework, as well as exploring barriers and facilitators to iART in a wide range of NYC clinics providing HIV primary care [22]. Given the flexibility in the definition of iART (e.g., starting ART on the same day as a reactive rapid test, starting ART after a lab-based confirmatory test), we sought to understand how iART is defined and clinic staff processes of providing iART to contribute to a better grasp of real-world applications of the approach. Understanding perceptions and uses of iART, as well as what is enabling and restricting its use, may enhance our ability to overcome implementation challenges, increasing our ability to bring iART to scale, and help inform efforts in other jurisdictions, with the goal of achieving better HIV outcomes and health equity more broadly [23].

## Methods

### Mixed methods design

We used a mixed methods, convergent study design to integrate results from a one-time, structured, internet-based quantitative survey and in-person qualitative in-depth interviews (IDIs) [24]. Several techniques were used to support data integration. First, all study instrument content (survey and IDI) was designed using the KAP framework to be complementary to support data linkage [22]. We used the KAP framework, which supports exploring each domain and their relationships, because knowledge, attitudes, and practices around iART may not be aligned given its flexible definition and less standardized protocols. Second, during data collection between January and April 2019, all participants who completed the survey were invited to complete an IDI, so the qualitative interviews serve as a sub-sample of the larger quantitative sample to strengthen the reliability of interpretation. Third, while we initially analyzed quantitative and qualitative data independently, we conducted regular meetings across researchers to share and discuss converges and divergences in preliminary and then later results, and to develop our integrated interpretation which we present in the discussion. Finally, we go back and forth between survey and IDI data throughout the methods, results and discussion to emphasize the use of combined data to construct this analysis.

### Study population and recruitment procedures

We selected a purposive sample of 30 NYC clinics that provide HIV primary care to people with HIV based on our ability to achieve a diverse cross-section believed to be representative of the larger clinic population [25]. To be eligible, clinics needed to meet the following criteria: (a) current provision of HIV primary care, (b) completed a clinic-based survey administered by NYC DOHMH in 2017, and (c) reported new HIV diagnoses in the previous 12 months above the median for all clinics that responded to the survey (median: 4, interquartile range [IQR]: 1–15). Current iART practices were not a criterion for selection. We dichotomized all eligible clinics at the median by percent of clients virally suppressed at the time of reporting (median: 84%, IQR: 77–87%), and selected 15 clinics from each group. We believe that many factors that influence viral suppression may also impact iART. Dichotomization helped us ensure that we selected a diverse range of clinics. We also conducted statistical tests to establish that significant differences did not exist between the groups by: (a) people with HIV caseload (i.e., people with established HIV and new HIV diagnoses), (b) clinic resources (e.g., availability of on-site pre-exposure prophylaxis [PrEP], post-exposure prophylaxis [PEP], pharmacy, number of medical providers), (c) clinic location (i.e., Manhattan vs. other borough [i.e., Bronx, Brooklyn, Queens, or Staten Island]), and (d) clinic type (i.e., hospital-based vs. community-based clinic).

We recruited at least one medical and one non-medical staff member from each selected clinic to participate in the quantitative survey. We defined medical staff as anyone certified to provide medical care (e.g., physicians, nurse practitioners) and non-medical staff as anyone providing non-medical or administrative services (e.g., case managers, clinic managers, social workers). We selected staff using an established contact list and publicly available data. Selected staff were sent recruitment emails to complete the internet-based survey, hosted on a secure server by SurveyGizmo (Boulder, CO, US). We obtained informed consent from all participants. Participants who completed the quantitative survey were invited to opt into the qualitative IDI. Survey respondents who expressed interest were contacted to schedule an IDI. We aimed, though did not require, that interviews represented dyads of a medical and non-medical staff from the same clinic.

### Quantitative survey measures

We asked participants to report the following: (a) staff position, (b) years of experience working in HIV, (c) acceptance of new patients for HIV primary care, (d) rapid HIV test availability, (e) on-site pharmacy availability (See Supplementary Material 1, Additional File 1). Additionally, we used data collected from the 2017 clinic-based survey to assess: (a) clinic location, (b) clinic type, (c) and clinic patient demographics (i.e., proportion of patients by race or ethnicity). All participants provided informed consent prior to taking the survey.

We assessed knowledge around the association of iART with VS and patient retention, as well as the association of VS with the elimination of sexual transmission of HIV, after defining iART as the following: “S*ame-day ART [or immediate initiation of ART] is defined as ART initiation on the same day as: HIV diagnosis or re-engagement in HIV care after a period of absence. When initiating same-day [or immediate] ART, antiretroviral medication may be given prior to receiving results of a confirmatory HIV test and baseline laboratory tests (e.g., basic metabolic panel [BMP], comprehensive metabolic panel [CMP], complete blood count [CBC], HIV genotype test, CD4 T lymphocyte cell count [CD4 count], plasma HIV RNA [viral load]).”* We measured attitudes by assessing respondent agreement with initiation of ART under various scenarios including initiation: (a) the same day as a reactive rapid HIV test, (b) within three to four days of a reactive rapid HIV test, (c) only after confirmatory HIV test results, (d) only after baseline laboratory test results (i.e., BMP, CMP, CBC), (e) only after genotype laboratory test results, or (f) only after all other HIV laboratory test results (i.e., CD4 count, viral load). We measured practice by the self-reported duration between a patient obtaining a reactive rapid test and the initiation of ART. While those diagnosed through laboratory testing are also capable of engaging in iART, we chose to measure practice based on a reactive rapid test because it provides staff a unique opportunity to immediately respond with treatment initiation. Additionally, clinic- and patient-related facilitators and barriers to provision of iART were assessed. We provided respondents a list of factors that could influence their ability to implement iART, as well as a free-response option. We asked respondents to select all applicable factors.

### Qualitative in-depth interview procedures

The IDI topic guide, designed to complement the survey, asked open-ended questions about iART knowledge, attitudes, and clinical practices with a focus on its implementation (See Supplementary Material 2, Additional File 2). Pre-determined probes explored contextual factors influencing iART implementation, with an emphasis on potential barriers and facilitators. Interviewees were also invited to elaborate upon issues they felt were pertinent but had not been pre-identified by the interviewers. Interviews were designed to last approximately one hour, were conducted by non-DOHMH researchers, and participants received a $50 gift card as a thank you for their time. Written consent was obtained prior to conducting each interview. Interviews were digitally recorded and professionally transcribed verbatim for analysis.

### Quantitative statistical analyses

We restricted quantitative analyses to eligible participants, who consented, and completed a survey. We included “not sure” responses in our analyses around iART knowledge; for all other analyses, we excluded individuals if they indicated that they preferred not to answer or were not sure. We grouped together responses based on agreement or disagreement in our analyses around attitudes (i.e., we grouped responses of strongly agree with agree and grouped strongly disagree with disagree). We generated descriptive statistics for quantitative survey respondents related to staff characteristics (e.g., years of HIV-related work experience) and clinic characteristics (e.g., hospital-based vs. community-based setting). We analyzed survey responses on an individual-level opposed to clinic-level since knowledge, attitudes, and practices, especially in the absence of formalized practice, are representative of an individual respondent and not the clinic. Separate binomial logistic regression models were fit to estimate odds ratios (OR) and 95% confidence intervals (95% CI) for the association between individual- and clinic-level respondent characteristics and (a) knowledge, attitudes, and practices around iART, and (b) barriers and facilitators to implementing iART. Results were considered significant at alpha equal to 0.05. All analyses were conducted in SAS 9.4 (SAS Institute; Cary, NC).

### Qualitative analysis

We used thematic analyses structured using a Framework Model [26]. The Model guided the development of an initial codebook based on pre-selected domains of knowledge, attitudes, and practices from the survey and complementary content areas from the IDI topic guide [26]. For example, we developed five codes related to the knowledge of the participants about iART, encompassing what was known and where it was learned. Two members of the study team independently read two transcripts and developed initial codes through discussion. They subsequently reviewed and mutually agreed upon the fit of the codes and confirmed them by analyzing two additional transcripts, with final adjustments of codes and definitions occurring as needed. The finalized codebook was applied to the remaining transcripts and a re-coding of the initial four transcripts. Codes were reviewed and summarized by theme. The team conducted analyses at the individual level with interviewees representing themselves, not their respective clinics. Our analysis around formalized clinic-level iART policies was the exception to this general rule. In keeping with the Framework Model we analyzed the data by comparing codes by cases and case groupings (i.e., knowledge of medical providers versus administrators). The team used descriptive analytic memos to describe these results. The entire study team reviewed the overall analysis and contributed to the interpretation.

### Ethics approval

The study was performed in accordance with the Declaration of Helsinki. Ethical approval was conducted by the Institutional Review Boards at the New City Department of Health and Mental Hygiene (IORG0000420, 45 CFR § 46.110[b][1][i]) and New York Psychiatric Institute (IORG0000275, 45 CFR § 46.110 [b][1][f]). Participants consented prior to taking part in the survey and in-depth interview. Informed consent was obtained from all the participants.

## Results

Among the 93 people we sent recruitment emails, 46 completed the survey (49%), representing 27 clinics with 15 of them supplying both a medical and non-medical provider dyad. Subsequently, 17 completed an IDI (37%), representing 12 clinics with 5 of them supplying both a medical and non-medical provider dyad. The interviews lasted on average 45 min, and took place in staffs’ HIV clinics (N = 14), by phone (N = 2), and in a quiet coffee shop (N = 1). The median length of time survey respondents reported employment in the field of HIV was 12.5 years (interquartile range [IQR]: 6–20 years), and 13.0 (IQR:9–20) years for IDI respondents. All clinics were accepting patients for HIV primary care, and the majority reported that rapid HIV testing and a pharmacy were available on-site (Table 1).

**Table 1:** Individual and clinic characteristics^a^ among study participants

	N
**Survey respondents**	
**Completed a survey**	46
**Years of HIV-related work, median (IQR)**	12.5 (6–20)
**Staff Position**	
Medical	20
Non-medical	26
**Clinic services**	
Accepting new clients	46
Rapid HIV testing available	39
On-site pharmacy	24
**Clinic type**	
Community-based clinic	25
Hospital-based clinic	21
**Clinic client population**	
Majority POC^b^	28
Majority non-POC^b^	14
**Clinic Borough**	
Manhattan	19
Other^c^	27
**In-depth interview participants**	
**Completed an in-depth interview**	17
**Years of HIV-related work, median (IQR)**	13.0 (9–20)

^a ^Clinic characteristics are presented for each respondent rather than each clinic to align with the unit of analysis in our study

^b ^POC (people of color) includes clients who identify as Asian, American Indian/Alaskan Native, Black/African American, Hispanic/Latino, Native Hawaiian/Other Pacific Islander, or identify with more than one race

^c ^Other boroughs include: the Bronx, Brooklyn, Queens, and Staten Island

### Knowledge

Among all survey respondents, 98% reported prior knowledge of iART, with high knowledge around VS and patient retention benefits (Table 2a and 2b). Differences in HIV knowledge did exist, with respondents from clinics located within Manhattan more likely to identify that people with an undetectable viral load cannot transmit HIV to sexual partners compared to respondents from other boroughs (OR: 7.9, 95% CI: 1.5–41.0). In the IDIs, non-medical staff demonstrated less familiarity with the concept beyond the idea that ART should be made available quickly compared to medical interviewees. Interviewees also differed on the procedural steps associated with iART. Some described iART as prescribing ART on the same day as a reactive rapid HIV test, while others identified needing confirmatory HIV test results before prescribing. Regardless of the specified HIV test, most interviewees viewed iART as starting ART as soon as possible, usually specifying same or next day starts. Across all provided definitions, interviewees mentioned that early treatment was associated with reduced time to VS, and improved patient retention and ART adherence, and described it as an opportunity to form a bond with patients, with one medical staff member (ID7) stating:

*I think the amount of attention and concern that a patient who’s getting immediate or same-day treatment… I think that means something to people. I think people walk away feeling like, I was taken care of and I was a priority today, because they were. I think that that will help to retain the person in treatment.*

**Table 2a:** Knowledge, attitudes, and practices around immediate initiation of antiretroviral therapy (iART)

	Overall % (n)	Staff Position			Clinic Patient Demographics			
**KAP Responses by Category**		Medical (%)	Non-Medical (%)	Medical vs. Non-medical OR (95% CI)	Majority POC^a^ (%)	Majority non-POC^a^ (%)	Majority POC^a^ vs. Majority non-POC^a^ OR (95% CI)	
**Knowledge**								
*Identified same-day ART as*:								
Decreasing time to viral load suppression	86.4 (38)	100.0	75.0	---	85.2	85.7	1.0 (0.2-6.0)	
Increasing patient retention	82.2 (37)	90.0	76.0	0.4 (0.06-2.0)	85.2	78.6	1.6 (0.3–8.3)	
*Identified undetectable viral load as*:								
Eliminating sexual transmission of HIV	67.4 (31)	70.0	65.4	0.8 (0.2–2.8)	57.1	85.7	0.2 (0.04–1.2)	
**Attitudes** ^**b,c**^								
*Agree with initiating ART*:								
Same day as a reactive rapid result	83.3 (35)	89.5	78.3	2.4 (0.4–13.8)	84.0	84.6	1.0 (0.2–6.1)	
Within 3–4 days of a reactive rapid result	69.8 (30)	79.0	62.5	2.3 (0.6–8.9)	68.0	71.4	0.9 (0.2–3.6)	
*Agree with initiating ART only after*:								
Confirmatory HIV results	51.2 (21)	**31.6**	**68.2**	**0.2 (0.06–0.8)** ^**b**^	52.0	41.7	1.5 (0.4–6.1)	
Baseline laboratory results	19.5 (8)	10.5	27.3	0.3 (0.06–1.8)	28.0	7.7	4.7 (0.5–42.9)	
HIV genotype laboratory results	7.3 (3)	5.0	9.5	0.5 (0.04-6.0)	8.0	0.0	---	
All other baseline HIV laboratory results	17.5 (7)	5.0	30.0	0.1 (0.01–1.1)	16.7	16.7	1.0 (0.2–6.4)	
**Practices** ^**b**^								
*Typical time to ART initiation*:								
Same day as reactive rapid test result	20.0 (8)	21.1	20.1	1.1 (0.2–5.3)	**8.7**	**38.5**	**0.2 (0.02-1.0)** ^**b**^	
Within 3–4 days of reactive rapid test result^d^	72.5 (29)	79.0	66.7	1.9 (0.5–7.8)	73.9	69.2	1.3 (0.3–5.7)	
≥ 5 days of reactive rapid test result	27.5 (11)	21.1	33.3	0.5 (0.1–2.2)	26.1	30.8	0.8 (0.2–3.6)	

OR = odds ratio; 95% CI = 95% confidence interval

^a^ POC (people of color) includes clients who identify as Asian, American Indian/Alaskan Native, Black/African American, Hispanic/Latino, Native Hawaiian/Other Pacific Islander, or identify with more than one race ^b^ p-value < 0.05

^b^ Survey respondents who selected not sure are excluded

^c^ Each timepoint or laboratory result was assessed as an individual question

^d^ Includes respondents who selected “same day as a reactive rapid test result”

**Table 2b:** Knowledge, attitudes, and practices around immediate initiation of antiretroviral therapy (iART)

	Overall % (n)	Clinic Type			Clinic Borough		
**KAP Responses by Category**		Hospital (%)	Community (%)	Hospital vs. Community OR (95% CI)	Manhattan (%)	Other^a^ (%)	Manhattan vs. Other OR (95% CI)
**Knowledge**							
*Identified same-day ART as*:							
Decreasing time to viral load suppression	86.4 (38)	85.0	87.5	0.8 (0.1–4.5)	79.0	92.0	0.3 (0.05-2.0)
Increasing patient retention	82.2 (37)	79.2	87.5	0.5 (0.1–2.2)	79.0	84.6	0.7 (0.2–3.2)
*Identified undetectable viral load as*:							
Eliminating sexual transmission of HIV	67.4 (31)	61.9	72.0	0.6 (0.2–2.2)	**89.5**	**51.9**	**7.9 (1.5–41.0)** ^**b**^
**Attitudes** ^**c,d**^							
*Agree with initiating ART*:							
Same day as a reactive rapid result	83.3 (35)	79.0	87.0	0.6 (0.1–2.9)	82.4	84.0	0.9 (0.2–4.6)
Within 3–4 days of a reactive rapid result	69.8 (30)	61.9	77.3	0.5 (0.1–1.8)	72.2	68.0	1.2 (0.3–4.6)
*Agree with initiating ART only after*:							
Confirmatory HIV results	51.2 (21)	57.9	45.5	1.7 (0.5–5.7)	55.6	47.8	1.4 (0.4–4.7)
Baseline laboratory results	19.5 (8)	15.8	22.7	0.6 (0.1–3.1)	25.0	16.0	1.8 (0.4–8.3)
HIV genotype laboratory results	7.3 (3)	5.3	9.1	0.6 (0.1–6.7)	0.0	12.5	---
All other baseline HIV laboratory results	17.5 (7)	21.1	14.3	1.6 (0.3–8.3)	12.5	20.8	0.5 (0.09–3.2)
**Practices** ^**c**^							
*Typical time to ART initiation*:							
Same day as reactive rapid test result	20.0 (8)	16.7	22.7	0.7 (0.1–3.3)	29.4	13.0	2.8 (0.6–13.8)
Within 3–4 days of reactive rapid test result^e^	72.5 (29)	66.7	77.3	0.6 (0.2–2.4)	76.5	69.6	1.4 (0.3–5.9)
≥ 5 days of reactive rapid test result	27.5 (11)	33.3	22.7	1.7 (0.4–6.9)	23.5	30.4	0.7 (0.2–2.9)

OR = odds ratio; 95% CI = 95% confidence interval

^a^ Other boroughs include: the Bronx, Brooklyn, Queens, and Staten Island

^b^ p-value < 0.05

^c^ Survey respondents who selected not sure are excluded

^d^ Each timepoint or laboratory result was assessed as an individual question

^e^ Includes respondents who selected “same day as a reactive rapid test result“

### Attitudes

In our assessment of individual timepoints for ART initiation, survey respondents agreed that ART should be initiated on the same day or within three to four days of a reactive rapid HIV test, with 83.3% and 69.8% agreeing, respectively (Table 2a and 2b). This sentiment was supported by survey findings where more than 80% of respondents agreed that ART initiation should not wait for baseline lab test results (i.e., BMP, CMP, CBC), HIV genotype test results, or all other baseline HIV test results (i.e., CD4 count, viral load). Conversely, 51.2% of survey respondents agreed that ART should only be initiated after receiving confirmatory HIV test results; compared to medical respondents, non-medical respondents were more likely to hold this belief (OR: 0.2, 95% CI: 0.06–0.8). Interviewees who advocated to start after confirmatory test results cited concerns around false positives, with some expressing unease prescribing ART only to later stop it in the case of a false positive test, for psychological as well as practical and economic reasons. Survey respondents reflected this, with 37% reporting false positives as a challenge to implementing iART. Other interviewees described how iART conveyed to patients the ‘urgency of viral control,’ and conferred a sense of importance to taking ART, with one medical provider (ID17) stating:

*I do think that there is something about immediate starts and that association that I think improves adherence… [The patient knows] that this is an important step and that adherence is important and that we care about you and that you know there’s no real reason to delay.*

Interviewees in general described a ‘different world’ of ART, given its tolerability, few side effects, low likelihood of resistance, and the new option of taking ART without an HIV diagnosis introduced by PrEP and PEP.

### Practices

Among all survey respondents, 73% reported zero to four days as the typical length of time from a reactive rapid HIV test to ART initiation for those patients who initiate, with 20% of respondents indicating same-day ART initiation as typical (Table 2a and 2b). Survey respondents from clinics serving a majority people of color (POC) were significantly less likely to report meeting the same-day benchmark (OR: 0.2, 95% CI: 0.02–1.0, p-value < 0.5). Interviewees described the importance of being ready to offer iART to clients, with one medical provider (ID4) stating:

*I think [iART] should always be offered… We don’t want to turn anyone away. If they’re ready for a service and we can provide it, we want to offer it to them, because you don’t know when they’re going to come back ready for that service… You want to be ready for them.*

Clinic practices, such as “being ready,” were often guided by policies and protocols. The iART policies interviewees described ranged from formal, semi-formal, to no instituted protocols. Two of the twelve interviewees’ clinics identified iART as a formal policy, and prescribed ART after a reactive rapid HIV test. An additional four interviewees clinics described their policies as a work in progress with discretion left to medical providers to immediately prescribe ART after a rapid HIV test. Two more interviewees described their clinics as having ad-hoc practices of initiating ART as soon as possible after a confirmatory HIV test result. The remaining four interviewees said that their clinics supported iART; however, they did not describe a formalized policy or protocol but did require a confirmatory test before commencing treatment.

### Barriers and facilitators to iART implementation

Among survey respondents, 33% indicated lack of medical provider experience with iART as a barrier. Survey respondents from clinics located in Manhattan were significantly less likely to report this barrier (OR: 0.2, 95% CI: 0.06–1.00, p-value < 0.05); these clinics were also significantly less likely to report increased medical provider experience with iART as a facilitator (OR:0.2, 95% CI: 0.03–0.9). During the IDIs, some interviewees listed patient acceptance of an HIV diagnosis and readiness to start treatment as a potential barrier, while others described this as an outdated idea, emphasizing how accepting the diagnosis has become decoupled from prescribing ART. Nearly all interviewed providers thought that patients now present with greater readiness to start ART. Greater familiarity with ART, and its tolerability and low pill burden, as well as PrEP and PEP, which were viewed as normalizing treatment use, were thought by interviewees to contribute to increased readiness among patients. However, 52% of survey respondents reported patient education materials around iART as important facilitators to ready patients for initiation. Interviewees also appeared to mentally construct groups based on perceived readiness to start treatment. For example, MSM who socialized with people taking ART or PrEP, or those seeking PrEP themselves, were viewed as more ready for iART, while individuals with substance use or mental health challenges, and heterosexual individuals whose HIV diagnosis “shocked” them were described as less ready for iART. Some interviewees disagreed with these groupings and expressed that iART should be universally applied.

Among survey respondents, 46% reported clinic-level financial barriers (e.g., medication cost due to challenges with same day insurance or pharmacy authorizations) as a challenge to iART; relative to respondents from other boroughs, respondents from clinics located in Manhattan were significantly more likely to report this as a barrier (OR: 8.0, 95% CI: 2.1–30.4). Survey respondents reported logistic barriers, including 76% reporting insurance barriers and 50% reporting prior authorizations as impeding iART (Table 3a and 3b). These barriers were also described in IDIs. At the time of the interviews, New York State was providing some facilities iART ‘coupons’ to immediately access one month of treatment prior to submission of a New York State Uninsured Care Programs (UCP) application. The UCP application is used to enroll clients in programs that cover the cost of United States Food and Drug Administration approved medications for low-income people with HIV who have coverage gaps (e.g., the AIDS Drug Assistance Program [ADAP]). Interviewees from clinics that did not have access to the ‘coupons’ cited UCP access delays, although several mentioned trainings and workarounds to expedite the process. Even with this support, quickly obtaining relevant paperwork and processing documentation for UCP access remained challenging for clinic staff. The degree of administrative effort around insurance coverage was reported as a significant factor shaping iART implementation; interviewees emphasized the importance of having experienced administrative staff and strong team-based approaches to facilitate the process of obtaining medications.

**Table 3a:** Barriers and facilitators to immediate initiation of antiretroviral therapy (iART)

	Overall % (n)	Staff Position			Client Patient Demographics		
		Medical (%)	Non-medical (%)	Medical vs. non-medical OR (95% CI)	Majority POC^a^ (%)	Majority non-POC^a^ (%)	Majority POC^a^ vs. Majority non-POC^a^ OR (95% CI)
**iART Clinic Barriers**							
Medication prior authorization	50.0 (23)	55.0	46.2	1.4 (0.4–4.6)	50.0	50.0	1.0 (0.3–3.6)
Financial barriers	45.7 (21)	50.0	42.3	1.4 (0.4–4.4)	42.9	57.1	0.6 (0.2–2.1)
Risk of false positive test	37.0 (17)	45.0	30.8	1.8 (0.6–6.2)	39.3	28.6	1.6 (0.4–6.5)
Lack of experience with same-day ART	32.6 (15)	30.0	34.6	0.8 (0.2–2.8)	32.1	42.9	0.6 (0.2–2.4)
Discomfort administering same-day ART	30.4 (14)	35.0	26.9	1.5 (0.4–5.2)	35.7	21.4	2.0 (0.5–9.1)
**iART Patient Barriers**							
Insurance barriers	76.1 (35)	75.0	76.9	0.9 (0.2–3.5)	85.7	64.3	3.3 (0.7–15.3)
Financial barriers	63.0 (29)	70.0	57.7	1.7 (0.5–5.9)	67.9	57.1	1.6 (0.4–5.9)
Psychosocial barriers	63.0 (29)	70.0	57.7	1.7 (0.5–5.9)	60.7	57.1	1.2 (0.3–4.3)
Patient refusal	53.2 (24)	60.0	46.2	1.8 (0.5–5.7)	46.4	50.0	0.9 (0.2–3.1)
Immigration status	47.8 (22)	45.0	50.0	0.8 (0.3–2.6)	50.0	42.9	1.3 (0.4–4.9)
**iART Facilitators**							
ART medication starter packs	63.0 (29)	65.0	61.5	1.2 (0.4–3.9)	75.0	50.0	3.0 (0.8–11.6)
Patient education materials	52.2 (24)	50.0	53.9	0.9 (0.3–2.8)	50.0	57.1	0.8 (0.2–2.7)
Financial support (e.g., grants)	47.8 (22)	45.0	50.0	0.8 (0.3–2.6)	53.6	35.7	2.1 (0.6–7.8)
Insurance navigation/enrollment staff	45.7 (21)	55.0	38.5	2.0 (0.6–6.4)	46.4	50.0	0.9 (0.2–3.1)
Increases appointment availability	39.1 (18)	35.0	42.3	0.7 (0.2–2.5)	35.7	42.9	0.7 (0.2–2.8)
Increased medical provider comfort	37.0 (17)	25.0	46.2	0.4 (0.1–1.4)	35.7	50.0	0.6 (0.2-2.0)
Increased case managers availability	34.8 (16)	45.0	26.9	2.2 (0.7–7.6)	28.6	35.7	0.7 (0.2–2.8)
Medication prior authorizations staff	34.8 (16)	35.0	34.6	1.0 (0.3–3.5)	42.9	28.6	1.9 (0.5–7.5)
Provider education materials	34.8 (16)	30.0	38.5	0.7 (0.2–2.4)	39.3	28.6	1.6 (0.4–6.5)
Patient psychosocial support services	30.4 (14)	35.0	26.9	1.5 (0.4–5.2)	32.1	21.4	1.7 (0.4–7.8)
Increased medical provider experience	28.3 (13)	25.0	30.8	0.8 (0.2–2.8)	32.1	28.6	1.2 (0.3–4.8)
Increased medical provider availability	21.7 (10)	30.0	15.4	2.4 (0.6–9.9)	21.4	14.3	1.6 (0.3–9.4)

OR = odds ratio; 95% CI = 95% confidence interval

^a^ POC (people of color) includes clients who identify as Asian, American Indian/Alaskan Native, Black/African American, Hispanic/Latino, Native Hawaiian/Other Pacific Islander, or identify with more than one race

**Table 3b:** Barriers and facilitators to immediate initiation of antiretroviral therapy (iART)

	Overall % (n)	Clinic Type			Clinic Location		
		Hospital (%)	Community (%)	Hospital vs. Community OR (95% CI)	Manhattan (%)	Other^a^ (%)	Manhattan vs. Other^a^ OR (95% CI)
**iART Clinic Barriers**							
Medication prior authorization	50.0 (23)	52.4	48.0	1.2 (0.4–3.8)	57.9	44.4	1.7 (0.5–5.6)
Financial barriers (e.g., medication costs)	45.7 (21)	42.9	48.0	0.8 (0.3–2.6)	**73.7**	**25.9**	**8.0 (2.1–30.4)** ^**b**^
Risk of false positive test	37.0 (17)	38.1	36.0	1.1 (0.3–3.6)	42.1	33.3	1.5 (0.4–4.9)
Lack of experience with same-day ART	32.6 (15)	28.6	36.0	0.7 (0.2–2.5)	**15.8**	**44.4**	**0.2 (0.06-1.0)** ^**b**^
Discomfort administering same-day ART	30.4 (14)	23.8	36.0	0.6 (0.2-2.0)	15.8	40.7	0.3 (0.06–1.2)
**iART Patient Barriers**							
Insurance barriers	76.1 (35)	71.4	80.0	0.6 (0.2–2.4)	79.0	74.1	1.3 (0.3–5.3)
Financial barriers (e.g., medication costs)	63.0 (29)	66.7	60.0	1.3 (0.4–4.5)	73.7	55.6	2.2 (0.6-8.0)
Psychosocial barriers	63.0 (29)	76.2	52.0	3.0 (0.8–10.6)	63.2	63.0	1.0 (0.3–3.4)
Patient refusal	53.2 (24)	61.9	44.0	2.1 (0.6–6.8)	47.4	55.6	0.7 (0.2–2.3)
Immigration status	47.8 (22)	42.9	52.0	0.7 (0.2–2.2)	47.4	48.2	1.0 (0.3–3.1)
**iART Facilitators**							
ART medication starter packs	63.0 (29)	**42.9**	**80.0**	**0.2 (0.05–0.7)** ^**b**^	57.9	66.7	0.7 (0.2–2.3)
Patient education materials	52.2 (24)	52.4	52.0	1.0 (0.3–3.2)	36.8	63.0	0.3 (0.1–1.2)
Financial support (e.g., grants)	47.8 (22)	47.6	48.0	1.0 (0.3–3.2)	47.4	48.2	1.0 (0.3–3.1)
Insurance navigation/enrollment staff	45.7 (21)	47.6	44.0	1.2 (0.4–3.7)	36.8	51.9	0.5 (0.2–1.8)
Increases appointment availability	39.1 (18)	33.3	44.0	0.6 (0.2–2.1)	31.6	44.4	0.6 (0.2-2.0)
Increased medical provider comfort	37.0 (17)	23.8	48.0	0.3 (0.1–1.2)	21.1	48.2	0.3 (0.1–1.1)
Increased case managers availability	34.8 (16)	28.6	40.0	0.6 (0.2–2.1)	36.8	33.3	1.2 (0.3-4.0)
Medication prior authorizations staff	34.8 (16)	23.8	44.0	0.4 (0.1–1.4)	36.8	33.3	1.2 (0.3-4.0)
Provider education materials	34.8 (16)	33.3	36.0	0.9 (0.3-3.0)	26.3	40.7	0.5 (0.1–1.9)
Patient psychosocial support services	30.4 (14)	33.3	28.0	1.3 (0.4–4.5)	21.1	37.0	0.5 (0.1–1.8)
Increased medical provider experience	28.3 (13)	14.3	40.0	0.3 (0.1–1.1)	**10.5**	**40.7**	**0.2 (0.03–0.9)** ^**b**^
Increased medical provider availability	21.7 (10)	19.1	24.0	0.8 (0.2–3.1)	10.5	29.6	0.3 (0.05–1.5)

OR = odds ratio; 95% CI = 95% confidence interval

^a^ Other boroughs include: the Bronx, Brooklyn, Queens, and Staten Island

^b^ p-value < 0.05

ART starter packs, consisting of a 7-day course of ART, often sourced from pharmaceutical companies, were the most frequently reported facilitator to alleviate logistic barriers, with 63% of survey respondents reporting it (Table 3a and 3b). Compared to survey respondents from community-based clinics, respondents from hospital-based clinics were significantly less likely to report ART medication starter packs as facilitators (OR: 0.2, 95% CI: 0.05–0.7). Several interviewees described starter packs as allowing immediate provision of ART, but concerns were expressed that logistical barriers to enable ongoing ART access were simply delayed. Interviewees described achieving success with iART using a multidisciplinary team-oriented approach, including non-medical staff skilled in insurance navigation, that followed their traditional approach to diagnosis and treatment but adjusted it to expedite ART delivery. Interviewees used different strategies, some describing a trial-and-error approach to find the right balance and order of operations involved in iART, from insurance enrollment to obtaining medication prior authorizations. One medical provider (ID4) described their experience navigating the demanding process:

*We had a PrEP patient come in… but when we rapid [HIV] tested him he was positive already. I thought, ‘Well, this is actually probably the perfect opportunity to try rapid start for this patient.’... We tried to actually start it that day, but logistic-wise, he ended up getting it if not the next day, that week for sure. We work with a few different pharmacies… so we tested the waters. We saw what works, what doesn’t work.*

Several emphasized the need for a coordinator as well as staff experience. Competing priorities were cited as a challenge to putting an iART process in place given their “huge caseloads,” expressed particularly by those interviewees who were less inclined to value same-day treatment initiation.

## Discussion

We found high levels of knowledge and acceptance of iART among survey respondents from HIV clinics in NYC, with initiation within three to four days of a reactive rapid HIV test more commonly practiced than same-day initiation. Significant differences did exist, with non-medical survey respondents more likely to agree that confirmatory HIV test results were needed before providing ART. It is important to ensure messaging is consistent across clinic staff. Inconsistent messaging has been shown to increase hesitancy around vaccinations, this may be translatable to ART [27]. Survey respondents from clinics serving a majority POC were less likely to report same-day ART initiation, and survey respondents from facilities in Manhattan were more likely to recognize that VS eliminates sexual HIV transmission. IDI analyses roughly mirrored these finding, while identifying specific contexts, conditions, and implementation practices. The interview analyses revealed that some of our pre-conceived survey measures around barriers and facilitators of iART were pertinent to interviewees, such as rapid versus confirmatory HIV tests, and insurance barriers. Further, they revealed salient issues not captured in the survey, including perceived patient readiness by providers. This is echoed in a survey among HIV providers in London where traditional views of patient readiness influenced provider willingness to start treatment [28]. We further found provider views on readiness by patient characteristics, which could potentially lead to disparities in access if certain groups were not viewed as “ready” for iART as other groups. Interview findings also suggested value in considering iART not as a separate intervention area, but as one that should be integrated with other initiatives within HIV clinics, such as PrEP and PEP, underscoring the importance of thinking of programs as interactive rather than discrete.

Data from the survey and interviews indicate that concerns remain around starting treatment prior to receipt of confirmatory HIV test results. These concerns are likely rooted in attitudes from the early days of the epidemic when medications were highly toxic and the potential negative consequences of needlessly starting a patient on treatment were great. HIV tests have improved in terms of sensitivity, specificity, and early detection [29]. Recommended initial ART regimens have favorable tolerability and toxicity profiles, making them approachable and easy to use for most newly diagnosed people [3, 30]. These newer regimens have demonstrated virologic efficacy and durability, supporting their immediate use for treatment, with the goal of rapid VS and improved individual and population health [3]. There is clear value in treating HIV immediately, like other infections, including the provision of patient-centric care and curtailing possible onward transmission [3, 6, 9]. Further, in the event of a false positive, iART provides a pathway to safely transition patients onto PEP or PrEP. A shift in HIV treatment practices to immediate treatment and integration with primary care, aligning with how other infectious diseases are managed, may continue to decrease HIV stigma, and increase perceived patient readiness for iART [31]. This will further drive down incidence and bring us closer to ending the epidemic.

Clinics need to have the infrastructure to immediately provide medication to facilitate this transition. Logistic issues around rapid HIV test access, insurance, prior authorizations, and medication costs were widely reported as barriers to iART, both in the survey and interviews. Interviewees described the real-world burden of quickly mobilizing insurance access and lab results, which require significant time and effort on the part of already overstretched staff. This finding suggests that clinic staff may need assistance operationalizing iART, including restructured workflows or dedicated iART teams to coordinate access to ART. Implementation of iART workflows will be important in mitigating NYC population-level differences in insurance coverage, as POC residents are more likely to be uninsured than non-Hispanic White residents [32]. Concurrently, differences exist by NYC borough, with generally higher levels of coverage and lower levels of unmet health care needs in Manhattan compared to other boroughs [33]. Disparities in insurance coverage, like other health inequities, are entwined in structural racism and create unjust barriers to ART [34]. Our finding around same-day ART initiation among survey respondents from clinics serving a majority POC reflect these inequities. To improve ART access for uninsured New Yorkers, New York State’s UCP implemented new ADAP enrollment procedures, including initial verbal confirmation of client eligibility after application submission for immediate ADAP activation [35]. Although the new procedures do not ameliorate greater systemic oppression, they take a step towards creating more equitable access to iART. Outreach to clinics promoting the new enrollment procedures, focused on those with less resources, should be conducted to ensure wide-spread knowledge of this resource. Outreach would provide further opportunities to evaluate obstacles to iART. Alleviation of logistic barriers is needed to expand iART access, with a focus on distributing resources to clinics serving populations that have experienced intersecting forms of structural oppression, including racism.

### Study limitations

Our study included a sample of staff from HIV clinics throughout NYC, but results are not generalizable to all NYC HIV clinic staff. Study recruitment occurred via purposive sampling, which allowed for a diverse cross-section of staff to take part in the assessment, but limited result generalizability. Due to the complex health insurance system in the United States, barriers and facilitators may not be translatable to other jurisdictions [36]. We found significant differences in survey responses by clinic-level VS and racial composition of client populations, potentially biasing results. Additionally, we conducted multiple statistical tests which increased the likelihood that a type I error occurred, with differences reported where none are actually present. Our relatively small purposive sample size might have resulted in insufficient power to detected differences between groups, resulting in a type II error, or may have introduced selection bias. Our assessment of iART did not obtain all relevant information, and importantly, patient perspectives were not included. Additionally, all selected clinics did not have access to rapid HIV tests, presenting potential issues in the measurement of iART practice for some respondents. There is also the potential for response bias if respondents expressed viewpoints they believed to be favorable. The limitations outlined are not unique to our study; they characterize many assessments around knowledge, attitudes, and practices. Our study also had substantial strengths. We gathered quantitative data through an internet-based survey that allowed for a geographically diverse sample, which, in comparison to other systems of data collection, might have also resulted in more honest responses and reduced potential response bias. Additionally, non-DOHMH researchers conducting interviews potentially mitigated response bias related to a desire to report positive opinions of iART and other activities supported by NYC DOHMH. Furthermore, our inclusion of a mixed methods design contextualized and enriched survey data and led to emergent and unexpected findings.

## Conclusion

As new HIV infections continue to decline it is important to ensure that progress is equitable across population groups. Our study found that improvements in implementation resources are needed to facilitate iART access for people newly diagnosed with HIV. If these resources are allocated and operationalized thoughtfully, current barriers may be overcome, and widespread adoption of iART made possible.

### Electronic supplementary material

Below is the link to the electronic supplementary material.

Supplementary Material 1

Supplementary Material 2

## Data Availability

The data and material are not available as they are stored on secured servers and cannot be shared. Reasonable requests can be submitted to the corresponding author for consideration by coauthors and Institutional Review Boards.

## Abbreviations

ADAP: AIDS Drug Assistance Program
ART: antiretroviral treatment
BMP: basic metabolic panel
CBC: complete blood count
CD4 count: CD4 T lymphocyte cell count
CI: confidence intervals
CMP: comprehensive metabolic panel
DOHMH: Department of Health and Mental Hygiene
iART: rapid or immediate initiation of ART
IDI: in-depth interview
KAP: knowledge, attitudes, and practices
MSM: men who have sex with men
NYC: New York City
OR: odds ratios
PEP: post-exposure prophylaxis
POC: people of color
PrEP: pre-exposure prophylaxis
UCP: Uninsured Care Programs
Viral load: plasma HIV RNA
VS: viral suppression
