# Supplementary material for: Immediate initiation of antiretroviral treatment: knowledge, attitudes, and practices among clinic staff in New York City
Source: BMC Health Serv Res. 2023 Sep 28;23:1039. doi: 10.1186/s12913-023-09896-5 (PMC10537909; doi:10.1186/s12913-023-09896-5)
Supplement: Supplementary file 1 — Supplementary Material 1 [file 12913_2023_9896_MOESM1_ESM.docx]

**Supplemental Material 1**

Quantitative survey tool used during data collection in the assessment of knowledge, attitudes, and practices, including barriers and facilitators, around immediate initiation of antiretroviral treatment among clinic staff in New York City.

| **Question** | **Responses** |
| --- | --- |
| What roles do you serve in your facility? Check all that apply. | - Clinical Provider (e.g., Physician, Nurse Practitioner, Physician Assistant) - Non-clinical Provider (e.g., Social Worker, Patient Navigator, Case Manager, Prevention Specialist) - Administrator (e.g., Practice Manager, Administrative Coordinator, Administrative Assistant, Billing Specialist) - Not sure - I prefer not to answer |
| What type of clinical provider are you? | - Internal Medicine Physician (MD/DO) - Family Medicine Physician (MD/DO) - Infectious Disease Physician (MD/DO) - Nurse Practitioner - Physician Assistant - Other (Please Specify:__________________________) - Not sure - I prefer not to answer |
| Approximately how many years, including time in training, have you been providing HIV care? | ___ ___ years   - Not sure - I prefer not to answer |
| What type of non-clinical role do you have? | - Social Worker - Patient Navigator - Case Manager - Certified Peer - Prevention Specialist - Other (Please Specify:__________________________) - Not sure - I prefer not to answer |
| Approximately how many years have you been providing HIV supportive care? | ___ ___ years   - Not sure - I prefer not to answer |
| What type of administrative role do you have? | - Practice Manager - Administrative Coordinator - Administrative Assistant - Billing Specialist - Other (Please Specify:__________________________) - Not sure - I prefer not to answer |

| Approximately how many years have you been working in an HIV clinic? | ___ ___ years   - Not sure - I prefer not to answer |
| --- | --- |
| Does your facility have a functioning onsite pharmacy? | - Yes - No - Not sure - I prefer not to answer |
| Is your facility currently accepting new patients living with HIV? | - Yes - No - Not sure - I prefer not to answer |

Thank you for sharing information about yourself and your facility. In the following section, we are interested in your level of agreement or disagreement related to statements about antiretroviral therapy (ART). Please provide your response to each item.

| **Question** | **Responses** |
| --- | --- |
| ART should be initiated on the same day as a reactive rapid test result. | - Strongly agree - Agree - Disagree - Strongly Disagree - Not sure - I prefer not to answer |
| ART should be initiated within 3-4 days of a reactive rapid test result. | - Strongly agree - Agree - Disagree - Strongly Disagree - Not sure - I prefer not to answer |
| ART should be initiated only after obtaining confirmatory HIV tests | - Strongly agree - Agree - Disagree - Strongly Disagree - Not sure - I prefer not to answer |
| ART should be initiated only after obtaining results of baseline laboratory tests (i.e., basic metabolic panel [BMP], comprehensive metabolic panel [CMP], complete blood count [CBC]). | - Strongly agree - Agree - Disagree - Strongly Disagree - Not sure - I prefer not to answer |
| ART should be initiated only after obtaining results of baseline HIV genotype laboratory tests. | - Strongly agree - Agree - Disagree - Strongly Disagree - Not sure - I prefer not to answer |
| ART should be initiated only after obtaining results of all other baseline HIV laboratory tests (i.e., CD4 T lymphocyte cell count [CD4 count], plasma HIV RNA [viral load]). | - Strongly agree - Agree - Disagree - Strongly Disagree - Not sure - I prefer not to answer |

Same-day ART is defined as ART initiation on the same day as: HIV diagnosis or re-engagement in HIV care after a period of absence.

When initiating same-day ART, antiretroviral medication may be given prior to receiving results of a confirmatory HIV test and baseline laboratory tests (e.g., basic metabolic panel [BMP], comprehensive metabolic panel [CMP], complete blood count [CBC], HIV genotype test, CD4 T lymphocyte cell count [CD4 count], plasma HIV RNA [viral load]).

| **Question** | **Responses** |
| --- | --- |
| Prior to this survey, had you heard about same-day ART? | - Yes - No - Not sure - I prefer not to answer |
| Would you say that same-day ART: | - Decreases the time until viral suppression - Increases the time until viral suppression - Does not impact the time until viral suppression - Not sure - I prefer not to answer |
| Would you say that same-day ART: | - Decreases patient retention - Increases patient retention - Does not impact patient retention - Not sure - I prefer not to answer |
| Would you say that having a consistently undetectable viral load for six months: | - Eliminates sexual transmission of HIV - Decreases but does not eliminate sexual transmission of HIV - Increases sexual transmission of HIV - Does not impact sexual transmission of HIV - Not sure - I prefer not to answer |

Thank you for sharing information pertaining to your thoughts and feelings about same-day ART. In the following section we are interested in your opinions about same-day ART. Please provide your response to each item.

| **Question** | **Responses** |
| --- | --- |
| Which of the following do you think are **challenges for your facility** in the initiation of same-day ART? Check all that apply. | - None - Lack of appointment availability - Lack of case manager availability - Lack of HIV testing on-site - Lack of clinical guidelines - Risk of false positive HIV test - Time to test results (e.g., BMP, CMP, CBC, HIV viral load, HIV genotype tests) - Medication prior authorization - Medical providers are too busy - Medical provider lack of experience with same-day ART - Medical provider discomfort administering same-day ART - Financial barriers (e.g., medication cost) - Other (**Please specify**: _______________________) - Not sure - I prefer not to answer |
| Which of the following do you think are **challenges for patients** in the initiation of same-day ART? Check all that apply. | - None - Financial barriers (e.g., medication cost) - Insurance barriers (e.g., uninsured patients) - Patient-related psychosocial barriers (e.g., patient fear, patient health literacy, patient perception) - Patient refusal (e.g., patient not interested, patient not ready to start ART) - Patient immigration status - Other (**Please specify**: _______________________) - Not sure - I prefer not to answer |
| Which of the following do you think would **support** having same-day ART administered by providers in **your clinic**? Check all that apply. | - None - Appointment availability - Case manager availability - HIV testing on-site - Clinical guidelines - Shorter time to test results - Dedicated staff for medication prior authorizations - Medical provider availability - Medical provider experienced with same-day ART - Medical provider comfort administering same-day ART - Financial support (e.g., grants, incentives) - Dedicated staff for insurance navigation/enrollment - Patient-related psychosocial support services - Patient education materials specific for same-day ART - Provider education materials specific for same-day ART - Same-day ART medication starter pack - Other (**Please specify**: _______________________) - Not sure - I prefer not to answer |

Thank you for sharing information pertaining to your opinions about same-day ART. In the following section please use the sliding scale to indicate where your opinions and beliefs fall as they relate to the time of antiretroviral (ART) initiation. Please provide your response to each item.

| **Question** | | **Responses** | | | | | |
| --- | --- | --- | --- | --- | --- | --- | --- |
| Among patients **not** living with HIV, during the following appointment types, what kind of HIV testing is available? Check all that apply. |  | Rapid testing | Laboratory testing | No testing available | Not applicable (appointment type not available) | Not sure | I prefer not to answer |
|  | Routine physical exams | 🞎 | 🞎 | 🞎 | 🞎 | 🞎 | 🞎 |
|  | Sexually transmitted infection (STI) screenings (symptomatic) | 🞎 | 🞎 | 🞎 | 🞎 | 🞎 | 🞎 |
|  | STI screenings (asymptomatic) | 🞎 | 🞎 | 🞎 | 🞎 | 🞎 | 🞎 |
|  | PrEP/PEP follow-up appointments | 🞎 | 🞎 | 🞎 | 🞎 | 🞎 | 🞎 |
|  | Gynecological appointments | 🞎 | 🞎 | 🞎 | 🞎 | 🞎 | 🞎 |
|  | Preventative care appointments (e.g., vaccination appointments) | 🞎 | 🞎 | 🞎 | 🞎 | 🞎 | 🞎 |
|  | Urgent care appointments | 🞎 | 🞎 | 🞎 | 🞎 | 🞎 | 🞎 |
|  | Adolescent care appointments | 🞎 | 🞎 | 🞎 | 🞎 | 🞎 | 🞎 |

| **Question** | **Responses** |
| --- | --- |
| Generally, among newly diagnosed patients, what is the average length of time between a reactive rapid test result and initiating ART? | - Same day - 1-4 days - 5-7 days - 8-14 days - Between 15 days **and** less than 1 month - Between 1 month **and** less than 2 months - Between 2 months **and** less than 3 months - 3 or more months - Not Sure - I prefer not to answer |

Thank you for sharing information pertaining to linkage to care at your clinic. We would like to ask you about your interest in taking part in an in-depth interview and for any referrals for this questionnaire.

| **Question** | **Responses** |
| --- | --- |
| We would like to conduct in-depth interviews with individuals about same-day ART. These interviews will focus on your beliefs, practices, knowledge, and perceived barriers and facilitators to same-day ART. Are you interested in being contacted about an in-depth interview? | - Yes - No |

\
