# Supplementary material for: Immediate initiation of antiretroviral treatment: knowledge, attitudes, and practices among clinic staff in New York City
Source: BMC Health Serv Res. 2023 Sep 28;23:1039. doi: 10.1186/s12913-023-09896-5 (PMC10537909; doi:10.1186/s12913-023-09896-5)
Supplement: Supplementary file 2 — Supplementary Material 2 [file 12913_2023_9896_MOESM2_ESM.docx]

**Supplemental Material 2**

**In-Depth Semi-Structured Interview Guide**

This semi-structured interview guide is designed as a list of lead questions the interviewer will ask, with follow-up probes associated with each question. Due to the semi-structured nature of the interviews, the probes will be explored by the interviewer in a flexible manner. Probes may not need to be covered based on the interviewees’ responses, their order may change, some may emerge as critical, while still other areas of discussion may arise that were not predicted in advance. Researchers are trained to establish rapport with interviewees to foster a substantial dialogue covering significant ground for each question asked.

1. Please tell me about your role at your organization.

- Facility and specific role
- Length of time at facility and in the HIV services field
- Training background

1. Please walk me through the process of patient diagnosis, linkage to care, prescription of ART, and follow-up at your clinic.

- Model overall service process (focusing on linkage, prescribing, and viral load suppression)
- Staff involved in delivery
- Differences between standard and rapid service delivery if broached

1. What kinds of benchmarks, standards or guidelines do you use for linking patients to care and prescribing ART?

- Internal processes for movement of patient through (e.g.) the Care Continuum Dashboard or other measures/cut-offs, etc.
- How do these guidelines/standards shape the delivery of timely ART?

1. What challenges do you face in reaching these goals for linkage and timely prescribing of ART?

- Factors: Institutional like resources or staff shortages; Structural like insurance, ADAP, etc.; Patient-level, like their perceptions limiting timely linkage and prescription
- All factors evenly weighted or are some more/less challenging than others?
- How do factors affect who gets linked, how, and when?

1. [If it has not yet arisen]: We are interested in your facility’s use of same-day initiation of ART for newly diagnosed patients. What do you know about same-day initiation of ART in New York or elsewhere?

- Knowledge of global, national, or local efforts in rapid initiation
- Knowledge of national/international recommendations for rapid treatment
- When did you first hear about same-day ART initiation?
- How have you seen “immediate” or “rapid” initiation defined? (Same day, one week, within 30 days?)
- How would you define “timely” linkage and “rapid” initiation? Both ideally and realistically for your facility

1. Does your agency have the capacity to implement same-day ART initiation?

IF Yes:

- Please provide brief history of adopting and implementing this strategy.
- At this point, how does it differ from the standard process for linkage and prescription of ART you previously described?
  - Populations engaged
  - Alterations to previous flow listen for use of ‘best practices’ from survey
- Reflecting on the immediate ART definition you provided earlier, is this the definition your agency also uses? If not, how does it differ?
- Please walk us through the workflow/process you use for same-day ART. Probe differences with ‘standard’ process. Particularly listen for use of tests, insurance, handoffs, relationships with other facilities, and overall use of ‘best practices’ from survey
- From your perspective, what is the outcome/outcomes of same-day ART initiation? Have you documented any clinical outcomes?
- Have you experienced any challenges or barriers in delivering? Of what kinds? [providers; patients; operations; structures; etc.]
- On the other side, are there strategies you have used to implement successfully or overcome initial barriers? Please provide a few examples.
- How are patients reacting to being offered same-day ART initiation?
- What, if any, additional resources do you think your agency needs for same-day ART?

1. If you do not yet do same-day initiation, please comment on the feasibility of introducing same-day ART at your organization.

- Feasibility as related to what kinds of internal/external factors? Issues related to patients or staff?
- What do you think needs to happen for same-day ART initiation to be adopted?

1. In your opinion, is same-day initiation of ART and timely linkage to care a central issue for HIV providers and patients in New York City?

- How does same-day initiation compare to other issues?

1. What sorts of additional resources should be devoted to scaling up same-day initiation?

- State/city resources, organization resources, or both?
- Value/importance of rapid initiation

10. We have covered a lot of ground today. Is there anything else you would like to talk about before our interview ends?
